# Development efficiency and mortality after coronary artery bypass grafting: a national causal inference analysis

**DOI:** 10.1186/s13019-026-04337-w

**Published:** 2026-06-08

**Authors:** Gabriel K. Martins, Arthur D. Botelho, Leo Consoli, Iago T. Grillo, Felipe S. Passos, Ricardo E. Treml, Tulio Caldonazo

**Affiliations:** 1 Faculty of Medicine, Medical Education Institute, Alagoinhas, Brazil; 2 Faculty of Medicine, Central University of Paraguay, Ciudad del Este, Paraguay; 3 School of Medicine, Federal University of Bahia, Salvador, Brazil; 4 University of Buenos Aires, Buenos Aires, Argentina; 5 Department of Thoracic Surgery, Hospital MaterDei, Salvador, Brazil; 6 Department of Anesthesiology, Perioperative and Pain Medicine, Stanford University, Stanford Medical School, Stanford, CA USA; 7 Department of Cardiothoracic Surgery, Jena University Hospital, Am Klinikum 1, Jena, 07747 Germany

**Keywords:** CABG, Human development index, Mortality, Causal mediation analysis

## Abstract

**Background:**

We evaluated whether development efficiency, the component of the Human Development Index independent of GDP per capita and income inequality, is associated with in-hospital mortality after coronary artery bypass grafting (CABG) in Brazil, and whether this association is mediated by access to elective surgery.

**Methods:**

We conducted a retrospective ecological panel study using administrative data on CABG hospitalizations within the Brazilian Unified Health System from 2008 to 2024. State-year observations were linked to socioeconomic indicators. Development efficiency was defined as the residual of HDI after regression on GDP per capita and the Gini coefficient. Associations with in-hospital mortality were examined using volume-weighted multilevel models. Absolute causal effects and mediation through urgency status were estimated using g-computation and parametric causal mediation analysis.

**Results:**

The final analytic panel included 379 state-year observations. A 1–standard deviation increase in development efficiency was associated with a reduction in predicted in-hospital mortality from 6.8% to 5.7% (absolute risk reduction − 1.1% points; *p* < 0.001). At the population level, this corresponds to approximately one fewer observed death per 91 procedures in states with higher versus lower development efficiency. Mediation analysis indicated that 95.5% of the total effect was attributable to the natural direct effect, while only 4.5% was mediated through urgency status, with no significant indirect effect.

**Conclusions:**

Development efficiency is an independent and clinically meaningful determinant of survival after CABG in Brazil. Higher income-independent HDI performance is associated with substantial absolute mortality reductions, driven predominantly by direct system-level pathways rather than changes in urgency profile. Strengthening health-system efficiency and perioperative capacity may therefore yield meaningful gains in cardiac surgical outcomes.

**Supplementary Information:**

The online version contains supplementary material available at 10.1186/s13019-026-04337-w.

## Introduction

Coronary artery disease remains the leading cause of death worldwide, accounting for approximately 17.5 million deaths each year, with nearly 80% occurring in low- and middle-income countries [1]. Coronary artery bypass grafting (CABG) is the gold-standard treatment for complex multivessel disease and provides durable survival benefits in high-risk populations [2–7]. Despite its effectiveness, global access to cardiac surgery remains highly unequal. An estimated 75% of the world’s population lacks timely access to CABG when clinically indicated, largely due to constraints in infrastructure, workforce capacity, and health-system organization rather than clinical contraindications [1, 4, 6]. Marked differences in specialist availability further illustrate this imbalance, with low-income countries averaging approximately 0.04 cardiac surgeons per million inhabitants versus more than 7 per million in high-income settings [1, 8]. Consistently, population-level analyses show that surgical mortality is inversely associated with the Human Development Index (HDI), indicating that outcomes after cardiac surgery are shaped not only by patient risk but also by broader social and institutional conditions [1, 4, 7, 9]. 

However, important uncertainties remain regarding how development influences surgical survival. Most prior investigations of disparities in cardiac surgery outcomes have focused on macroeconomic indicators such as gross domestic product per capita or income inequality (the Gini coefficient) [10]. While useful, these measures do not capture how effectively societies convert available resources into health, education, and longevity [8, 10]. Notably, nearly one-third of the variation in HDI is independent of income and inequality, representing a dimension of development efficiency that may more directly reflect health-system performance [1, 10]. The relationship between this income-independent component of development and CABG mortality has not been formally evaluated using causal inference methods, and the mechanisms linking development to perioperative outcomes remain insufficiently characterized.

Brazil provides a uniquely informative setting to examine these relationships. The Brazilian Unified Health System (Sistema Único de Saúde, SUS) is one of the largest universal public health systems worldwide and offers nationwide coverage for cardiac surgery. Despite universal entitlement, the delivery of CABG varies by more than 700-fold across states, reflecting deep heterogeneity in surgical capacity, workforce distribution, and perioperative infrastructure [8, 11]. This combination of universal coverage with large regional variation creates a natural analytic framework to evaluate how development-related factors translate into differences in surgical outcomes within a single national system. In this study, we evaluated the hypothesis that the income-independent component of development efficiency is associated with lower in-hospital mortality after CABG in Brazil, and that this association operates predominantly through direct health-system mechanisms rather than solely through improved access to elective surgery [1, 10]. 

## Methods

### Data sources and study design

We performed a retrospective, population-based ecological panel study using administrative data on CABG hospitalizations within the Brazilian Unified Health System (SUS) from January 1, 2008, through December 31, 2024. Surgical data were obtained from the SUS Hospital Information System (SIH/SUS), which captures all publicly funded inpatient procedures nationwide.

### Study population and variables

CABG hospitalizations were identified using national procedure codes for coronary revascularization (04.06.01.092-7, 04.06.01.093-5, 04.06.01.094-3, 04.06.01.095-1). The outcomes included were in-hospital mortality and procedures volume.

Key covariates included revascularization extent, urgency status (proportion of urgent/emergency procedures), calendar year, and geographic macroregion (North, Northeast, Southeast, South, Central-West), modeled as a hierarchical level to account for regional clustering and health-system heterogeneity.

Hospitalization records were aggregated at the state-year level (27 states over multiple years) and stratified by procedure type and urgency status. This aggregation strategy allowed us to capture temporal and geographic variation in surgical volume and outcomes while preserving comparability across states within the public health system.

### Socioeconomic exposures

Socioeconomic indicators were derived from national public databases. HDI values were obtained from the Brazilian Institute of Geography and Statistics for the years 2012–2021 and interpolated within states for earlier years (2000–2011) using linear interpolation. Annual illiteracy rates (2012–2021); gross domestic product (GDP) per capita (2013–2016); and the Gini coefficient (2000 and 2010) were similarly interpolated to generate complete annual state-level data series, as complete data availability for those years was not possible from official public sources. To ensure temporal ordering, all socioeconomic exposures were lagged by one year relative to surgical outcomes, establishing temporal precedence and minimizing the potential for reverse causation.

### Development efficiency decomposition

To separate development performance from pure economic resources, lagged HDI was regressed on lagged GDP per capita and lagged Gini coefficient. The residual from this model, accounting for 28.6% of HDI variance (adjusted R² = 0.7137), was defined as development efficiency, representing the capacity of social and institutional systems to convert available resources into health, education, and longevity, independent of financial inputs.

### Statistical analysis

To account for heteroskedasticity and large inter-state variability in surgical volume (> 700-fold), state-year observations were volume-weighted to stabilize mortality estimates. Multilevel regression models were fitted with state-year as the analytic unit and macroregion as a higher-level grouping factor. Weights were defined as the number of CABG procedures in each state-year relative to the total study volume, so that states with higher-volume programs, with more stable mortality estimates, contributed proportionally more to model fitting and small-volume states had reduced influence from stochastic variation.

Absolute causal effects were estimated using g-computation with covariates held at observed values. Causal mediation analysis was conducted using quasi-Bayesian simulation (1,000 draws) to decompose total effects into natural direct and indirect components mediated by urgency proportion.

Sensitivity analyses included: (1) restriction to high-volume states (mean procedures ≥ 75th percentile); (2) elective-only procedures; (3) non-interpolated data only; and (4) alternative time periods. Spatial autocorrelation was assessed using Moran’s I. Robustness to unmeasured confounding was evaluated with E-values. Multicollinearity was checked via variance inflation factors (VIF), ensuring it was < 5 in all models.

All analyses were performed in R. Two-sided *P* < 0.05 was considered statistically significant, with emphasis on absolute effects and clinical interpretability given the rarity of mortality [1]. Absolute effects were calculated to enhance interpretability. The Absolute Risk Reduction (ARR) was estimated using g-computation by calculating the difference between the marginal predicted mortality at the baseline HDI residual and the predicted mortality following a one-standard deviation (1-SD) increase in development efficiency. To facilitate interpretation of effect magnitude, the inverse of the ARR (1/ARR) was computed as a descriptive metric representing the number of CABG procedures in a higher-efficiency ecological context that would correspond to one fewer observed death compared to a lower-efficiency context. This metric should be interpreted as a population-level summary of effect size within the ecological framework of this study, not as a patient-level clinical recommendation analogous to an NNT from a randomized trial.

### Handling of missing data and interpolation

Missing socioeconomic data was addressed via linear interpolation within states, ensuring a complete panel. Interpolation flags were tracked, and sensitivity analyses restricted to non-interpolated years showed consistent results.

### Ethical considerations

The study used only de-identified, aggregated public data from the SIH/SUS database. No individual-level identifiable information was accessed, and all analyses were conducted at the state-year level without direct involvement of human participants. In accordance with Brazilian regulations, studies based exclusively on anonymized secondary data from publicly available databases are exempt from Research Ethics Committee review, as established by National Health Council Resolution No. 510/2016.

## Results

### Study population

The final analytic dataset included 379 state-year observations of CABG hospitalizations in the SUS from 2008 to 2024, representing more than 96% of eligible CABG records after lagging and completeness filters (excluding state-years without procedures). Substantial heterogeneity was observed across states in both socioeconomic development and CABG outcomes.

Before presenting the primary causal estimates, it is important to contextualize the observed administrative mortality figures. The unadjusted in-hospital mortality in this dataset (baseline 6.8% in 2008) is higher than the risk-adjusted rates reported in dedicated clinical registries from Brazil and other middle-income settings during the same period, which have generally ranged from 3% to 5% for isolated CABG. This discrepancy reflects several features inherent to administrative data: the SIH/SUS does not reliably distinguish isolated CABG from combined procedures (e.g., CABG + valve surgery), which carry substantially greater operative risk; case-mix adjustment for individual comorbidities, coronary anatomy, and operative complexity is not possible; and coding practices may vary across institutions and over time. These administrative mortality figures should therefore not be interpreted as representing the true risk-adjusted outcome of isolated CABG in Brazil, but rather as a composite indicator of CABG-related in-hospital events within the public health system. The primary analytical goal of this study is to examine the association between development efficiency and relative changes in this administrative mortality indicator across states and years, rather than to benchmark absolute mortality against clinical registry standards.

### Absolute causal effect

Using g-computation with parametric simulation–based causal mediation, a 1–standard deviation increase in development efficiency was associated with a reduction in predicted in-hospital mortality from 6.8% to 5.7%, corresponding to an absolute risk reduction of − 1.1% points (95% CI − 1.53 to − 0.61; *p* < 0.001; Table 1).

**Table 1:** Absolute Causal Effect of a 1-Standard Deviation Increase in HDI Residual on In-Hospital CABG Mortality (G-Computation)

Quantity	Estimate
**Baseline mortality (low HDI residual)**	6.8%
**Mortality after + 1-SD in HDI residual**	5.7%
**Absolute risk reduction**	−1.1% (95% CI: −1.53% to − 0.61%)
**P value**	< 0.001
**Ecological effect size (procedures per fewer observed death)**	91

Estimates from g-computation holding constant surgical risk factors, urgency mix, procedure type, calendar year, and regional structure. Absolute risk reduction interpreted as percentage points

**Fig. 1:**
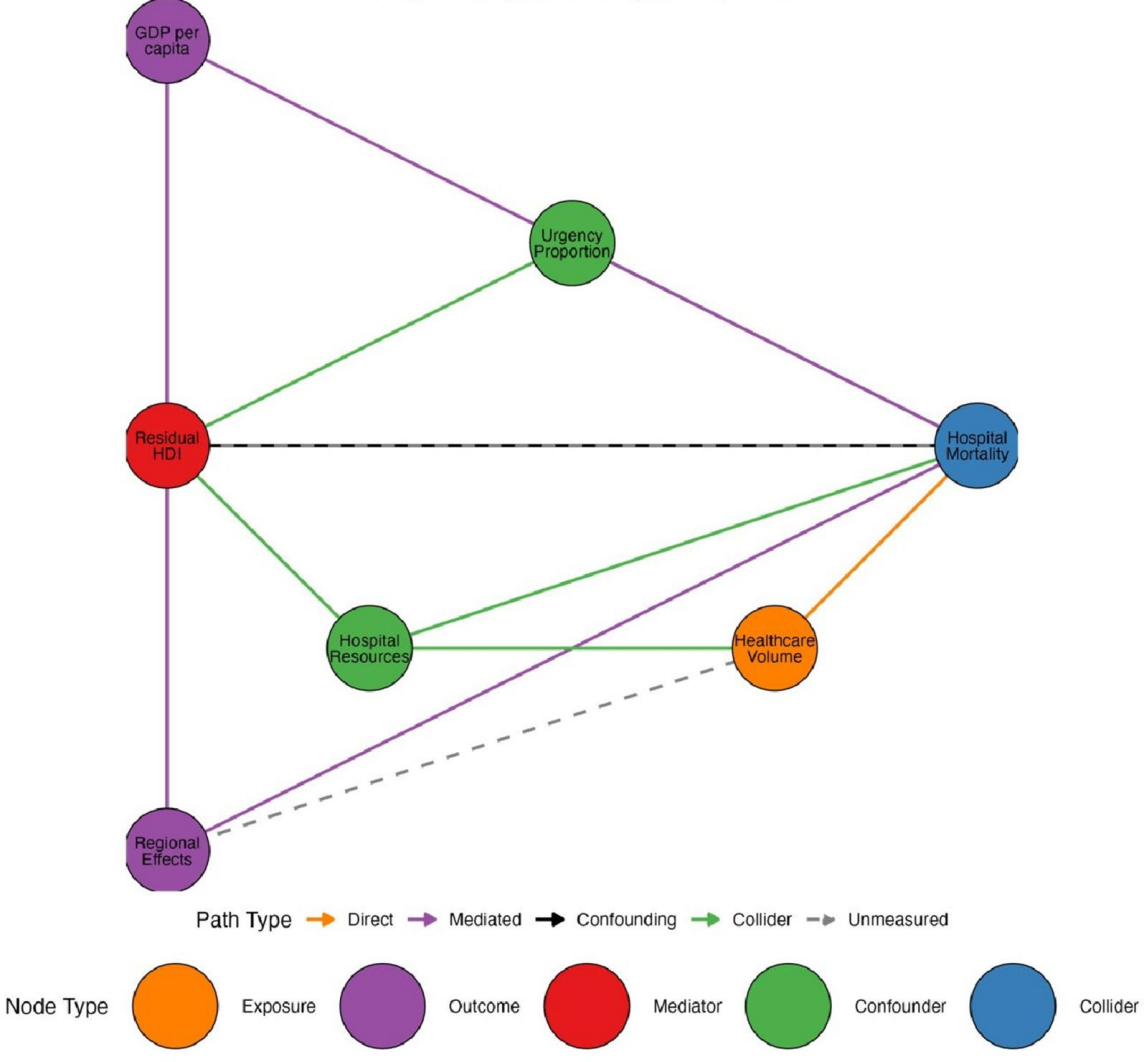
Directed Acyclic Graph of the Causal Pathways Between Development Efficiency (Residual HDI) and In-hospital Mortality Following Coronary Artery Bypass Grafting.This causal diagram maps the hypothesized relationships between socioeconomic exposures, system-level mediators, and surgical outcomes. Development Efficiency (Residual HDI), defined as the component of the Human Development Index independent of income and inequality, serves as the primary exposure. Urgency Proportion and Hospital Resources are identified as potential mediators through which development may influence survival. GDP per capita and Regional Effects are treated as confounding variables to account for economic resources and geographic health-system heterogeneity

Effect decomposition indicated that the natural direct effect accounted for most of the survival benefit, while the proportion of urgent procedures did not significantly mediate the association (Fig. 1; Table 2).

**Table 2:** Decomposition of the Effect of HDI Residual on CABG Mortality via Proportion of Urgent Procedures

Effect	Risk difference	95% confidence interval	*P* value
**Total effect**	−1.10%	−1.53% to − 0.61%	< 0.001
**Natural direct effect (NDE/ADE)**	−1.02%	−1.47% to − 0.58%	< 0.001
**Natural indirect effect (NIE/ACME)**	−0.05%	−0.16% to 0.01%	0.108
**Proportion mediated (average)**	4.5%	−7% to 15%	0.108

NDE = natural direct effect; NIE = natural indirect effect; ACME = average causal mediation effect. Mediation via proportion of urgent CABG procedures. Estimates from parametric simulation-based approach (quasi-Bayesian)

### Mediation by surgical urgency

Mediation analysis showed that the natural direct effect accounted for nearly all the observed benefit (− 1.02%, 95% CI − 1.47% to − 0.58%; *p* < 0.001), whereas the natural indirect effect through urgency proportion was small and not statistically significant (− 0.05%, 95% CI − 0.16% to 0.01%; *p* = 0.108). The proportion mediated was 4.5% (95% CI − 7% to 15%), indicating that most of the total effect was direct rather than mediated by urgency status.

### Clinical and population-level implications

**Fig. 2:**
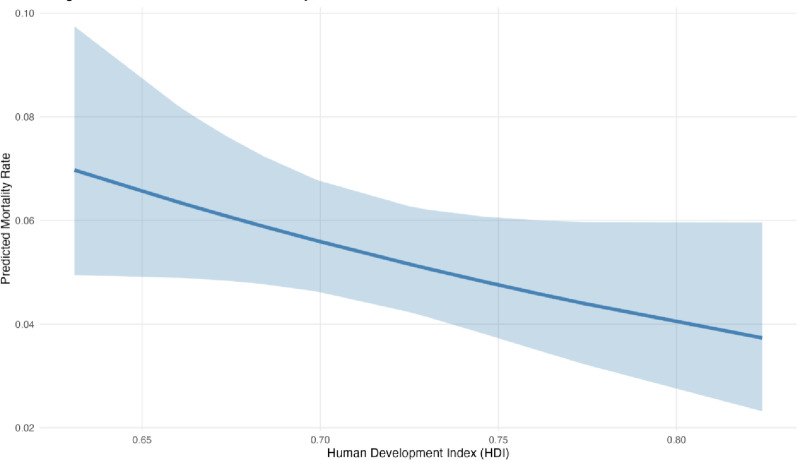
Marginal Effect of the Human Development Index on Predicted CABG Mortality Rate.This figure illustrates the predicted probability of in-hospital mortality following coronary artery bypass grafting (CABG) as a function of the Human Development Index (HDI). The solid blue line represents the marginal predicted mortality rate, showing a clear inverse relationship where higher development levels are associated with lower mortality. The shaded area indicates the 95% confidence interval. According to the predictive model, a 1-standard deviation increase in development efficiency leads to a significant absolute reduction in mortality from 6.8% to 5.7%

At the national level, given approximately 40,000 annual CABG within SUS, a 1-SD increase in development efficiency would be expected to prevent roughly 440 in-hospital deaths per year (Fig. 2).

### Robustness of the association

The association remained stable across sensitivity analyses, including restriction to high-volume centers, elective procedures only, and alternative time-period specifications. No meaningful spatial autocorrelation was detected (Moran’s I = − 0.002; *p* = 0.31). The E-value was 1.66 for the point estimate and 2.26 for the lower confidence bound, indicating that an unmeasured confounder would need to be more strongly associated with both development efficiency and mortality than most known clinical risk factors to fully explain the observed effect.

## Discussion

In this nationwide longitudinal ecological analysis of CABG within the Brazilian SUS, higher development efficiency, measured as the income-independent component of the HDI, was associated with lower in-hospital mortality. Each 1-SD increase in this measure corresponded to an absolute 1.1% reduction in administrative mortality, which at the ecological level translates to approximately one fewer observed in-hospital death per 91 procedures when comparing higher- versus lower-efficiency states, and approximately 440 fewer deaths annually at the national level if all states were to achieve a 1-SD improvement. This effect size is substantial and comparable in magnitude to that of major therapeutic advances in cardiovascular care [12], though caution is warranted in direct comparisons given the ecological nature of the present study.

More than 95% of the observed survival benefit was not mediated by reductions in urgent surgery. Although urgent CABG is associated with substantially higher mortality [13, 14], the effect of development efficiency appears to operate predominantly through direct system-level mechanisms. This interpretation is supported by evidence that institutional experience, perioperative organization, ICU staffing, and structured quality programs are major determinants of cardiac surgical outcomes [11–17]. High-volume centers show 13–15% lower mortality than low-volume hospitals [18], and intensivist-led cardiac ICUs and formal quality-improvement programs reduce postoperative mortality by 30–50%, independent of surgical timing [19–21]. Together, these data indicate that survival depends more on perioperative system capacity and organization than on urgency status alone.

The concept of development efficiency as operationalized here—the residual of HDI after partialling out GDP per capita and the Gini coefficient—is an empirically derived construct rather than a pre-established index validated in the social science literature. Several points regarding its interpretation are warranted. First, residual-based constructs are inherently model-dependent: the magnitude and distribution of the residual depends on the specification of the regression model used to remove economic and inequality components. In this study, GDP per capita and the Gini coefficient explained 71.4% of HDI variance (adjusted R² = 0.7137), leaving 28.6% as the residual, which is consistent with published estimates suggesting that roughly one-third of HDI variation is income-independent. Second, the residual captures variance in HDI not explained by income and inequality, but it does not directly measure any single social or institutional domain—it is a composite signal reflecting unmeasured efficiency-related dimensions such as educational attainment independent of wealth, health-system organization, institutional quality, and social capital. Third, unmeasured confounders that are correlated with both the residual and surgical mortality could in principle drive the observed association. The E-value analysis (point estimate: 1.66; lower CI bound: 2.26) provides some quantitative bound on this concern, suggesting that an unmeasured confounder would need to be more strongly associated with both exposure and outcome than most established clinical risk factors to fully explain the observed association. Notwithstanding these caveats, the HDI residual approach is conceptually analogous to partial regression techniques used in comparative development economics to isolate the contribution of governance and institutional quality from income effects, and its application here is a reasonable and transparent operationalization of income-independent development performance. The HDI residual reflects dimensions such as education, institutional capacity, and health-system functionality that are not captured by income alone. Low health literacy, limited workforce density, and inadequate hospital preparedness are each independently associated with worse cardiovascular and surgical outcomes [22–28]. Regions with fewer than 20–40 surgical specialists per 100,000 inhabitants show substantially poorer surgical and obstetric results, and global estimates indicate that workforce expansion could avert hundreds of thousands of deaths annually [24–26]. At the hospital level, reliable infrastructure, medication availability, organized referral pathways, dedicated teams, and perioperative quality programs are consistently associated with lower mortality and failure-to-rescue rates, with reported reductions of up to 50% [20, 27–33]. Together, these system-capacity domains closely align with what is captured by the HDI residual.

Taken together, these findings carry important policy implications. In a middle-income country with universal health coverage, development efficiency functions as a structural determinant of cardiac surgical survival. Gains in this domain have the potential to save hundreds of lives annually after CABG by strengthening the system-level pathways identified in our mediation analysis. Evidence-based strategies include regionalization to high-volume centers [9, 11, 34, 35], minimum surgical workforce thresholds [24, 25, 35], structured perioperative quality programs such as ERAS and care bundles [31, 32, 35], health-literacy interventions to support recovery [22, 33], and expansion of dedicated, intensivist-led cardiac intensive care units [19, 20, 34]. Investing in these organizational and capacity-building measures offers a scalable route to improving surgical outcomes and links social and institutional development directly to cardiovascular survival.

### Study Limitations

This study has several important limitations. First, the ecological design introduces a risk of ecological fallacy, as associations observed at the state-year level may not fully reflect individual-level relationships. The g-computation and mediation analyses presented here rely on assumptions of exchangeability and no unmeasured confounding at the ecological level; because individual patient-level variables are absent, these assumptions cannot be formally verified. Results should therefore be interpreted as associations within an ecological framework rather than as individual-level causal estimates. Second, the absence of patient-level clinical variables is a major limitation. The SIH/SUS administrative data precludes adjustment for age, comorbidities (e.g., diabetes, renal failure, left ventricular function), coronary anatomy, operative complexity, and surgeon or center experience. The observed administrative mortality rates are therefore not equivalent to risk-adjusted clinical outcomes, and residual confounding by case-mix cannot be excluded. This limitation is further amplified by the inability to distinguish isolated CABG from combined procedures (e.g., CABG + valve surgery), which carry substantially higher perioperative risk and may be differentially distributed across states. Third, socioeconomic series required linear interpolation for several variables, particularly GDP per capita (2013–2016 only available) and the Gini coefficient (census years 2000 and 2010 only). Sensitivity analyses restricted to non-interpolated years showed consistent results, suggesting that interpolation did not materially alter conclusions, though temporal smoothing bias cannot be fully excluded. Fourth, changes in SIH/SUS coding practices over time may have affected procedure classification and outcome reporting. Fifth, volume weighting was used to account for the large inter-state variance in surgical volume; however, this approach concentrates analytical influence in high-volume states, which may differ systematically from low-volume states in unmeasured dimensions such as infrastructure, referral patterns, and patient selection. While this weighting strategy improves stability of mortality estimates, it may introduce selection-related bias. Unweighted sensitivity analyses were not formally presented but represent an important robustness check; readers should note that the weighting strategy may limit generalizability of findings to smaller or more peripheral states. Sixth, urgency proportion was selected as the sole mediator in the mediation analysis as a proxy for access to elective surgery. However, this represents an intentionally narrow mediating pathway. Other plausible system-level mediators—including ICU capacity, intensivist staffing, surgeon experience, hospital volume, availability of perioperative quality programs, and patient travel distance to care—were not included. The finding that most of the effect is attributable to the natural direct effect should be interpreted with caution: it does not mean that urgency is the only relevant pathway, but rather that urgency proportion, as modeled here, does not explain most of the observed association. Important unmeasured mediators almost certainly exist, and the residual direct effect likely represents a composite of multiple system-level mechanisms not captured in this analysis. Finally, differential reporting quality across states is possible, such that more developed systems may capture outcomes more completely, introducing potential information bias and endogeneity. Despite these constraints, the consistency of results across multiple sensitivity analyses and their concordance with the broader surgical outcomes literature support the robustness of the main findings.

## Conclusion

Development efficiency is an independent and clinically important determinant of survival after CABG in Brazil. Improvements in the income-independent component of HDI are associated with substantial absolute reductions in in-hospital mortality, largely through direct system-level pathways rather than shifts in urgency status. Investments in health-system efficiency and perioperative capacity are therefore central to improving cardiac surgical outcomes.

## Supplementary Information

Below is the link to the electronic supplementary material.

Supplementary Material 1.

Supplementary Material 2.

## Data Availability

The datasets analyzed during the current study are available in the DATASUS repository (Information System of the Unified Health System - SIHSUS), accessible at https://datasus.saude.gov.br/. Socioeconomic indicators were obtained from the Brazilian Institute of Geography and Statistics (IBGE) and the Atlas of Human Development in Brazil. All data used are publicly available, anonymized, and do not require administrative permissions for access. The R code and the processed datasets used for causal inference and mediation analysis are available from the corresponding author on reasonable request.
